# SOX9 mediates the phenotypic transformation of vascular smooth muscle cells in restenosis after carotid artery injury

**DOI:** 10.3389/fcell.2025.1592594

**Published:** 2025-06-18

**Authors:** Chuan Jiang, Jiasen Ye, Jie Huang, Yang Gao, Hong Chen, Fuqiang Guo, Lei Guo, Xiaofan Yuan

**Affiliations:** ^1^ Department of General Practice, Sichuan Provincial People’s Hospital, School of Medicine, University of Electronic Science and Technology of China, Chengdu, China; ^2^ Department of Neurosurgery, Southwest Medical University, Luzhou, Sichuan, China; ^3^ Department of Neurology, Sichuan Provincial People’s Hospital, School of Medicine, University of Electronic Science and Technology of China, Chengdu, China; ^4^ Department of Neurology, Xindu District People’s Hospital of Chengdu, Chengdu, China

**Keywords:** SOX9, signal transducer and activator of transcription 3, vascular smooth muscle cells, transformation, proliferation, migration, restenosis, carotid artery injury

## Abstract

In-stent restenosis (ISR) remains a significant public health challenge globally, as millions of stents are implanted annually. Elucidating the mechanisms underlying ISR is essential for developing effective preventive and therapeutic strategies. In this study, we identified SOX9, a transcription factor, as a key factor involved in the pathogenesis of ISR. Morphological and histological analyses of human carotid atherosclerotic plaques revealed high expression of SOX9 at the interface between the fibrous cap (FC)—predominantly composed of α-smooth muscle actin (α-SMA)-positive vascular smooth muscle cells (VSMCs)—and the lipid-rich necrotic core (LRNC), enriched with CD68-positive macrophages. This region is characterized by a high frequency of phenotypic transformation of VSMCs. Using a carotid artery balloon injury model, we observed high expression of SOX9 in the neointima, and SOX9 knockdown significantly attenuated intimal hyperplasia. *In vitro*, SOX9 knockdown in primary VSMCs suppressed platelet-derived growth factor-BB (PDGF-BB)-induced phenotypic transformation, proliferation, and migration. Further studies using CUT&Tag analysis indicated that PDGF-BB promotes the AMPK signaling pathway, leading to the nuclear translocation of SOX9. A dual-luciferase reporter assay revealed that SOX9 directly binds to the motif of the signal transducer and activator of transcription 3 (STAT3) promoter, thereby enhancing the phenotypic transformation of VSMCs. This study uncovered a novel molecular mechanism in which AMPK-mediated SOX9 activation facilitates its interaction with STAT3 to regulate the transformation, proliferation, and migration of VSMCs. These findings suggest that targeting the SOX9–STAT3 axis can serve as a promising therapeutic strategy for the prevention and treatment of ISR.

## Background

According to the global burden of cardiovascular diseases (CVDs) and risks from 1990 to 2022, ischemic heart disease ranks as the leading cause of global age-standardized disability-adjusted life years among all diseases (Mensah et al., 2023a). Although the mortality rate of CVD decreased by 34.9% from 358.4 per 100,000 in 1990 to 233.2 per 100,000 in 2022, the number of deaths attributed to CVD increased from 12.4 million in 1990 to 19.8 million in 2022 due to factors such as global population growth and aging (Mensah et al., 2023b). Percutaneous coronary intervention (PCI), as the primary method for the prevention and treatment of CVD, has remarkably advanced over the past 40 years. Initially, balloon angioplasty was developed, followed by the deployment of bare-metal stents (BMSs) for PCI. More recently, drug-eluting stents (DESs) and bioresorbable stents (BRSs) have been increasingly applied in clinical practice (Giustino et al., 2022; Piccolo et al., 2015). Although DESs have significantly reduced the incidence of restenosis compared to BMS, in-stent restenosis (ISR) and the need for repeated revascularization can be observed in approximately 1%–2% of patients annually (Madhavan et al., 2020; Moussa et al., 2020). Stent implantation induces mechanical injury to the endothelium and vascular wall, triggering the proliferation and migration of vascular smooth muscle cells (VSMCs) and excessive production of the extracellular matrix (ECM), finally resulting in neointimal hyperplasia (Giustino et al., 2022; Hoffmann et al., 1996). Since millions of DESs, BMSs, and BRSs are implanted globally each year, ISR represents a pathologic entity with considerable public health importance. Therefore, understanding the mechanism underlying restenosis is critical for devising effective preventive and therapeutic strategies to address ISR.

Carotid balloon injury has been widely recognized as a well-established animal model for investigating ISR (Tulis, 2007a; Tulis, 2007b). This model exemplifies the complex adaptive vascular responses characterized by the phenotypic transformation of VSMCs from a quiescent and contractile state in the medial layer to a proliferative and synthetic phenotype. Activated VSMCs migrate into the intimal layer, contributing to neointimal hyperplasia. Simultaneously, enhanced synthesis of the ECM and increased secretion of pro-inflammatory cytokines drive pathological remodeling, which finally culminates in ISR (Poole et al., 1971; Maguire et al., 2017; Kumar and Lindner, 1997). Synthetic VSMCs constitute a significant portion of the structural composition of pathological vessel walls. Studies have reported significant vascular remodeling after 4 weeks of carotid artery ligation in rats, which is characterized by a decreased vascular diameter and neointima formation, leading to an approximately 80% reduction in the luminal area (Herring et al., 2017). Further analysis of the neointima revealed that the majority of its cellular components originated from previously differentiated medial VSMCs. Specifically, VSMCs labeled with myosin heavy chain 11 and α-smooth muscle actin (α-SMA) constituted 79% ± 17% and 81% ± 12% of VSMCs, respectively (Herring et al., 2014). Thus, elucidating the molecular mechanisms underlying the phenotypic transformation of VSMCs may pave the way for developing novel therapeutic strategies to prevent ISR.

SRY-related high-mobility group box gene 9 (SOX9), a member of the group E subgroup, is composed of four functional domains: two C-terminal transactivation domains, a dimerization domain, and a DNA-binding high-mobility group domain (Schepers et al., 2002; Jo et al., 2014). These domains enable SOX9 to regulate diverse biological processes, including cell differentiation, proliferation, and reprogramming. Initially identified in patients with campomelic dysplasia during chondrogenesis (Wagner et al., 1994), impaired SOX9 localization and expression have been linked to the development and progression of various diseases, particularly fibrotic diseases and cancer (Jo et al., 2014). In vascular diseases, the distinct biological behaviors of venous and arterial VSMCs, shaped by their respective microenvironments, play a critical role in disease progression. Among the key molecular regulators, SOX9 has emerged as a pivotal regulator of transplant arteriosclerosis, thus orchestrating the phenotypic transformation of VSMCs (Yu et al., 2022; Yu et al., 2018). This regulatory function is driven by its capacity to activate autophagy-dependent pathways (Yu et al., 2022) and competitively inhibit the interaction between myocardin and the serum response factor (Yu et al., 2018). Furthermore, the structure and composition of the ECM are vital determinants of VSMC phenotype and function. SOX9 has also been shown to modulate ECM remodeling and attenuate vascular calcification by inhibiting the osteogenic differentiation of VSMCs (Zhou et al., 2021; Augstein et al., 2018). In addition, SOX9 has a central role in vascular aging, acting through a self-reinforcing feedback mechanism (Faleeva et al., 2024). Cellular senescence and ECM stiffening upregulate SOX9 expression, which subsequently exacerbates ECM remodeling and accelerates cellular senescence, thereby expediting the progression of vascular aging. Studies (Chang et al., 2024; Han et al., 2021) have demonstrated that platelet-derived growth factor-BB (PDGF-BB) activates VSMCs by promoting the phosphorylation of signal transducer and activator of transcription 3 (STAT3), thereby facilitating VSMC proliferation and migration while downregulating apoptosis and cell death. Conversely, the inhibition of the STAT3 signaling pathway has been shown to effectively prevent the formation of neointimal lesions by suppressing VSMC proliferation (Daniel et al., 2012). However, the potential regulatory relationship between SOX9 and STAT3 and their role in mediating the phenotypic transformation of VSMCs and the progression of ISR remains unclear. Fe_3_O_4_ generated from iron stents was demonstrated to inhibit the proliferation of VSMCs and phenotypic transformation by downregulating SOX9, thereby attenuating neointimal hyperplasia (Deng et al., 2024), but the underlying mechanisms remain poorly understood. In addition, in the context of ISR, the intricate interplay between SOX9 function and STAT3 regulation warrants further exploration.

Therefore, the current study investigated the role of SOX9 in neointimal hyperplasia following balloon injury and explored the mechanisms underlying VSMC transformation, proliferation, and migration *in vivo* and *in vitro*. Elucidating possible interactions with STAT3 can help us understand the molecular mechanisms involved in ISR and identify novel therapeutic targets for its prevention and treatment.

## Materials and methods

### Human specimens

Human carotid artery specimens were obtained from patients undergoing carotid endarterectomy (CEA). All participants provided written informed consent for inclusion in this study, which was approved by the Ethics Committee of the Sichuan Provincial People’s Hospital (Chengdu, China).

### Carotid balloon injury

The carotid balloon injury surgery was performed as described previously (Bendeck et al., 1994; Cheng et al., 2017). Briefly, following complete anesthesia of the rats, a midline incision was made along the anterior cervical region to expose the left common carotid artery, internal carotid artery, and external carotid artery. Vascular clamps were applied to temporarily block the blood flow of the common and internal carotid arteries. A small incision was made in the external carotid artery using ophthalmic scissors, allowing for the insertion of a 2F balloon catheter, which was advanced into the common carotid artery. The balloon was inflated with saline until slight resistance was encountered, at which point it was rotated and withdrawn to denude the endothelium of the artery. This process was repeated three times. Upon completion, the catheter was removed, the external carotid artery was ligated, and the clamps were released to restore blood flow. In the control group, a sham operation was performed, identical to the abovementioned procedure but without the insertion of the balloon catheter into the carotid artery. In both groups, a 5-mm segment of the carotid artery proximal to the bifurcation of the common carotid artery was harvested at 7, 14, and 21 days post-operation for analysis.

### Carotid lentivirus intervention

As we previously described (Yuan et al., 2023), 50 μL of 30% F127 Pluronic gel (P2245; Sigma) containing 5 μL of either LV-shSOX9 (1 × 10^12^ TU/mL) or LV-negative control (NC, 1 × 10^12^ TU/mL) was coated around the bifurcation of the carotid artery in balloon injury and sham operation rats, respectively. Then, LV-shSOX9- and LV-NC-treated carotid arteries were harvested 14 days post-operation. The efficiency of LV-shSOX9 was evaluated by autofluorescence and Western blot analysis. LV-shSOX9 and LV-NC were kindly provided by Shuliang Guo of the First Affiliated Hospital of Chongqing Medical University.

### Hematoxylin and eosin staining

Hematoxylin and eosin (H&E) staining (G1076; ServiceBio) was performed to assess the histological structure of the carotid artery, following the established protocol (Yuan et al., 2023). To evaluate stenosis after balloon injury, the thickness of the carotid artery wall was measured vertically from the intima to the media in four directions on cross-sections using ImageJ software (National Institutes of Health). The average thickness was then calculated. A positive correlation was demonstrated between the average arterial thickness and the severity of vascular stenosis after balloon injury.

### Immunohistochemistry and immunofluorescence

Primary rat VSMCs and arterial sections were collected for immunofluorescence (IF) and immunohistochemistry (IHC), as we previously described (Yuan et al., 2023). They were used to assess the subcellular localization of SOX9 in VSMCs and its expression in human carotid advanced atherosclerotic plaques and balloon-injured carotid artery tissues, and to evaluate the role of SOX9 in VSMC phenotypic transformation and carotid artery restenosis following balloon injury. Samples were incubated with primary antibodies against SOX9 (1:100 dilution, ab185230, Abcam), α-SMA (1:200 dilution, ab7817, Abcam), and CD68 (1:100 dilution, ab201340, Abcam) at 4°C overnight, followed by secondary antibodies such as Alexa Fluor 488 (1:200, SA00013-5, Proteintech, China) and 555 (1:100, A0460, Beyotime, China) for 1 h at room temperature. Nuclei were counterstained with DAPI (C1002, Beyotime, China). Images were captured using a confocal laser scanning microscope (Andor, United Kingdom) and analyzed using ImageJ software.

### Primary cell culture, treatment, and transfection

Primary VSMCs were isolated from the thoracic aortas of SD rats (male, weight: 100–150 g), as described previously (Kumar and Lindner, 1997). SM22α and α-SMA-positive cells were identified as VSMCs. The cells were cultured in Dulbecco’s modified Eagle medium (DMEM; 88,287, Gibco, United States) supplemented with 20% fetal bovine serum (FBS; C0235, Gibco, Australia), and they were used between passages 3 and 6 in subsequent experiments.

Small interfering RNAs (siRNAs) against SOX9 (si-SOX9) and NC (si-NC) were designed and synthesized by GenePharma (Shanghai, China). *In vitro* transfection with siRNAs was performed using Lipofectamine RNAiMAX Reagent (13778030, Invitrogen, United States), according to the manufacturer’s instructions. The Si-SOX9 sequences are as follows: CGCUCACAGUACGACUACATT (forward) and UGUAGUCGUACUGU GAGCGTT (reverse). The efficiency of SOX9 knockdown was verified by quantitative real-time PCR (qRT-PCR) and Western blot analysis.

VSMCs were divided into different groups as follows: (i) PDGF-BB (0, 10, and 20 ng/mL for 48 h and 10 ng/mL for 0, 24, and 48 h) group. PDGF-BB (10 ng/mL for 48 h) was used for follow-up experiments. (ii) si-NC, si-NC + PDGF-BB, si-SOX9, and si-SOX9 +PDGF-BB groups. (iii) DMSO, AMPK inhibitor, PDGF-BB, and PDGF-BB + AMPK inhibitor groups. Recombinant murine PDGF-BB and AMPK inhibitors (HY-151361, MCE) were purchased from PeproTech (Rocky Hill, NJ, United States) and MCE (Shanghai, China), respectively.

### Cell proliferation and migration assay

The proliferation and migration abilities of VSMCs were evaluated using 5-ethynyl-20-deoxyuridine (EdU, C0085, Beyotime, China), a Cell Counting Kit-8 (CCK8, C0037, Beyotime, China), and a wound-healing assay, following previously described methods (Yuan et al., 2023; Wu et al., 2019) and the manufacturers’ protocols. For the CCK8 assay, VSMCs were transfected with si-SOX9 or si-NC, then seeded into a 96-well plate and cultured in serum-free medium overnight. The cells were incubated with or without 10 ng/mL PDGF-BB for 48 h, after which 10 μL of solution was added to each well, followed by incubation at 37°C for 2 h. Optical densities (ODs) at 450 nm were measured using a microplate reader (Heales, China). For the EdU assay, VSMCs were seeded into 24-well plates and cultured in DMEM supplemented with or without 10 ng/mL PDGF-BB and si-SOX9. Then, 10 μM EdU was added for 2 h, and the cells were fixed in 4% paraformaldehyde for 15 min. Subsequently, the cells were treated with 0.3% Triton X-100 at room temperature for 10 min and stained with DAPI. Cell proliferation was observed under a fluorescence microscope (Leica, United Kingdom). For the wound-healing assay, VSMCs were cultured in 6-well plates until approximately 80–90% confluence, treated as mentioned above, and then subjected to serum starvation for 24 h. The confluent cells were scratched using a 200 μL pipette tip. Cell migration of the scratched areas was monitored at 0 and 24 h after the assay using a bright-field microscope (Leica, Germany). The migration rate was analyzed as the ratio of the migrated area relative to the initial wound area.

### qRT-PCR

Total RNA was isolated from VSMCs using TRIzol reagent (TaKaRa, 9108), and 1 μg aliquots were reverse-transcribed into cDNA using a reverse transcription kit (TaKaRa, PR047). Quantitative real-time polymerase chain reaction was performed using SYBR Green reagent (TaKaRa, PR802A), according to the manufacturer’s protocols. The relative mRNA expression levels were normalized to β-actin.

### Western blotting

Total proteins were extracted from VSMCs or tissues using the RIPA lysis buffer (PM0013B; Beyotime, China) and quantified using a bicinchoninic acid protein assay kit (P0009; Beyotime, China). Then, equal amounts of protein were loaded onto SDS-PAGE gels, transferred to polyvinylidene fluoride membranes (Millipore, China), blocked with 5% non-fat milk, and incubated overnight at 4°C with the primary antibodies against SOX9 (1:1,000 dilution; ab185230; Abcam), α-SMA (1:5,000 dilution; ab7817; Abcam), SM22α (1:5,000 dilution; 10493-1-AP; Proteintech), α-tubulin (1:5,000 dilution; 14555-1-AP, Proteintech), STAT3 (1:1,000 dilution; 9139; CST), p-STAT3 (1:2,000 dilution; 9145; CST), AMPK (1:2,000 dilution; 5831; CST), and p-AMPK (1:1,000 dilution; 2535; CST). In addition, horseradish peroxidase-conjugated anti‐rabbit (1:5,000 dilution; SA00001‐2; Proteintech) or anti‐mouse (1:5,000 dilution; SA00001‐1; Proteintech) antibodies were used as secondary antibodies. Images were captured and quantified using the Fusion FX5 image analysis system (Vilber Lourmat). The gray value of the target gene, relative to α-tubulin, represented the relative protein level.

### CUT&Tag

Both the control group of VSMCs and those treated with PDGF-BB for 48 h were harvested and processed in accordance with the manufacturer’s protocol (Vazyme, TD904) and a previous study (Kaya-Okur et al., 2019). Briefly, the cells were permeabilized using the non-ionic detergent digitonin to increase membrane permeability. The permeabilized cells were then incubated overnight at 4°C with a primary antibody against SOX9 (dilution 1:1,000; ab185230; Abcam). On the following day, a secondary antibody (dilution 1:1,000; Proteintech, sa00001-2) was added and incubated for 1 h to form antibody–antigen complexes. Hyperactive pA/G transposon Pro was subsequently introduced to specifically bind these complexes. Upon activation, the transposase facilitated precise cleavage of DNA regions adjacent to the antibody-bound target protein. Following this, DNA was extracted, purified, and amplified via PCR. High-throughput sequencing was performed using the NovaSeq X Plus System (PE150, United States), and the resulting data were processed and analyzed to identify the targeted genomic regions.

### Dual luciferase reporter assay

The promoter sequences of STAT3 were subcloned into the pGL4 luciferase promoter–reporter vector (GenePharma). VSMCs were seeded into 96-well plates at a density of 5 × 10^4^ cells per well and co-transfected with the constructed luciferase reporter vectors and either pLV4-SOX9 or the empty pLV4 vector using an advanced transfection reagent, following the manufacturer’s protocol. After 48 h of incubation, the cells were lysed, and firefly luciferase activity was measured using a Dual-Luciferase Reporter Assay Kit (Catalog No. MA0520-1, MeilunBio), according to the manufacturer’s instructions. Firefly luciferase activity was normalized to Renilla luciferase activity to account for transfection efficiency.

### Statistical analysis

Statistical analysis was performed using GraphPad Prism 9.0 (GraphPad, United States) and SPSS 26.0 (IBM, United States) software. All data represent results from at least three independent experiments. Student’s t-test and one-way repeated measures analysis of variance (ANOVA) were used to compare differences between groups and subgroups. Chi-square and Mann–Whitney U tests were used for nonparametric data. A difference was considered statistically significant when p < 0.05.

## Results

### Carotid balloon injury upregulates SOX9 in the neointima

**FIGURE 1:**
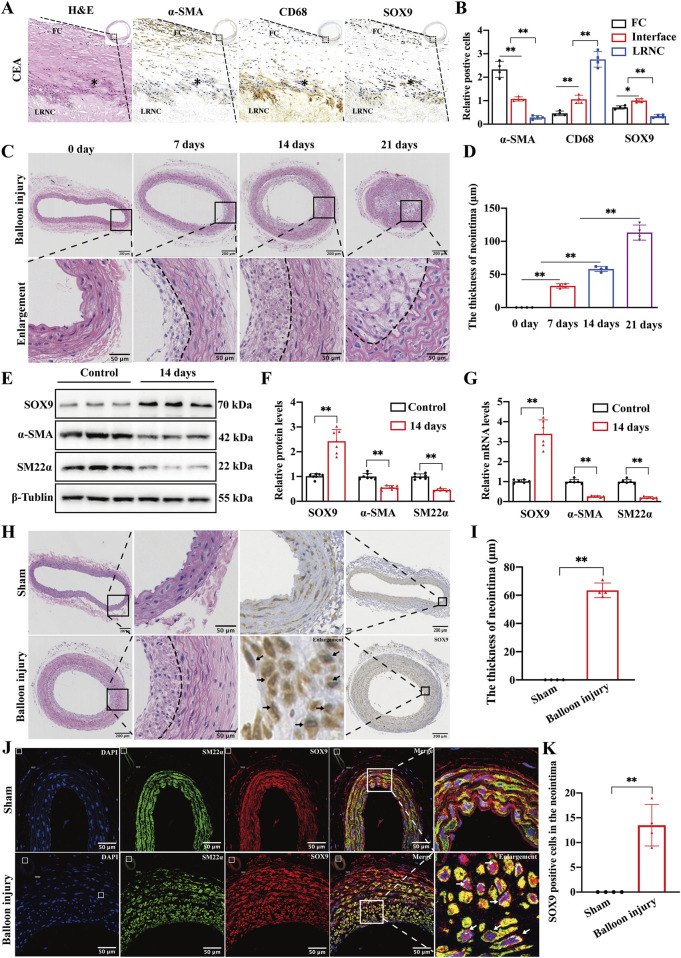
Carotid balloon injury upregulates SOX9 in the neointima **(A, B)** H&E and IHC staining of specimens obtained from CEA in patients diagnosed with severe carotid artery stenosis revealed that advanced atherosclerotic plaque is characterized by an LRNC beneath the FC **(A)**. The quantification of IHC **(B)** further demonstrated that the FC was predominantly composed of α-SMA-positive VSMCs, while the LRNC was infiltrated by CD68-positive macrophages. The expression of SOX9 was markedly elevated at the interface (*) between the outer layer of the LRNC and the medial layer of the FC, which is the region with a high frequency of VSMC phenotypic transformation during the formation and progression of atherosclerosis. The region of interest (ROI) represents the FC, and * indicates the interface between the medial layer of the FC and the outer layer of LRNC. CEA, carotid endarterectomy, FC, fibrous cap, LRNC, lipid-rich necrotic core. n = 4; *p < 0.05; **p < 0.01; scale bar, 1,000 μm and 100 μm. **(C,D)** Representative photomicrographs of H&E staining **(C)** and quantification **(D)** for the thickness of the neointima in the sham-operated and balloon-injured carotid arteries on days 7, 14, and 21. The degree of intimal hyperplasia exhibited a time-dependent escalation in proportion to the progression of vascular injury duration. n = 4; **p < 0.01; scale bar, 200 μm and 50 μm. **(E–G)** Western blot analysis **(E)** and RT-PCR showed the protein **(F)** and mRNA **(G)** levels of SOX9, α-SMA, and SM22α in the sham-operated and balloon-injured carotid arteries at 14 days post-injury. n = 6; **p < 0.01. **(H–K)** Representative photomicrographs of H&E and IHC staining in the sham-operated and balloon-injured carotid arteries at 14 days post-injury **(H)**. Histological analyses found increased SOX9 deposition in the neointimal lesions of balloon-injured carotid artery (black arrows). The thickness of the neointima increased in the balloon-injured group compared with that in sham-operated carotid arteries **(I)**. IF staining further confirmed that SOX9 was highly expressed in the VSMCs residing in the neointima of balloon-injured carotid arteries but not in VSMCs retained in the media of the vessel wall (white arrows, J). The quantification of SOX9-positive cells in the neointima of sham and balloon-injured carotid arteries showed obvious differences **(K)**. n = 4; **p < 0.01; scale bars, 200 μm and 50 μm.

We conducted morphological and histological analyses of specimens obtained from CEA in patients with carotid artery stenosis. H&E staining revealed that atherosclerotic lesions caused luminal stenosis, with advanced plaque characterized by a lipid-rich necrotic core (LRNC) beneath the fibrous cap (FC). IHC staining also demonstrated that the fibrous cap was predominantly composed of α-SMA-positive VSMCs, while the LRNC was infiltrated by CD68-positive macrophages. Notably, SOX9 expression was markedly upregulated at the interface between the outer layer of the lipid core and the medial layer of the fibrous cap (Figures 1A,B). This region is more likely to exhibit the phenotypic transformation of VSMCs during the development and progression of atherosclerosis, suggesting that SOX9 may play a critical role in regulating the phenotypic transformation of VSMCs in atherosclerotic plaques.

Following stent implantation and other procedures that mechanically damage the arterial intimal layer, VSMCs in the media undergo phenotypic transformation. In addition, these cells subsequently proliferate and migrate into the intima, playing a critical role in lumen restenosis. In this study, we employed the rat carotid balloon injury model to investigate the formation and regression of ISR. The thickness of the neointima gradually increased on days 7, 14, and 21 after balloon injury compared to that of sham-operated carotid arteries (Figures 1C,D). Based on these findings, we selected the 14-day model for subsequent experiments. Western blotting and qRT-PCR were conducted on the sham-operated arteries and arteries subjected to the 14-day post-balloon injury model. The results (Figures 1E–G) show that the protein and mRNA levels of SOX9 were increased, and VSMC markers (α-SMA and SM22α) were downregulated in post-balloon injury arteries. Furthermore, H&E and IHC staining demonstrated significant luminal restenosis in injured carotid arteries, accompanied by increased SOX9 expression in neointimal lesions (Figures 1H,I). The quantification of IF staining also confirmed that after balloon injury, SOX9 was highly expressed in VSMCs in the neointima but not in VSMCs retained in the media of the vascular wall (Figures 1J,K). Collectively, the co-localization of SOX9 expression with phenotypically transformed VSMCs migrating into the neointima highlights the role of SOX9 in driving neointimal hyperplasia and restenosis following carotid balloon injury.

### SOX9 expression is increased in VSMCs induced by PDGF-BB

**FIGURE 2:**
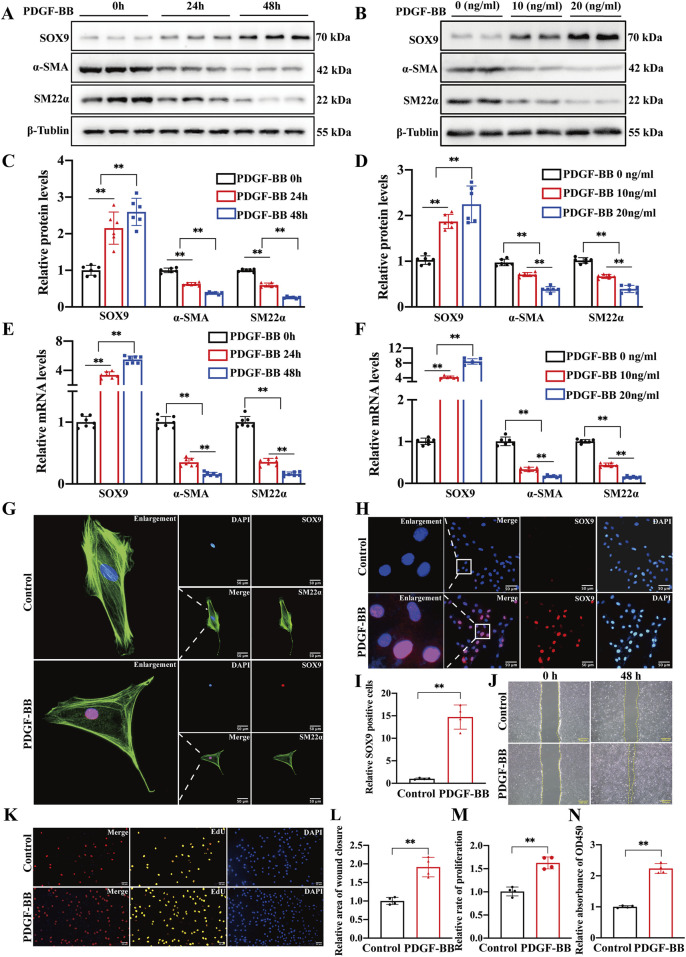
SOX9 expression is increased in VSMCs induced by PDGF-BB **(A–F)** Western blot analysis **(A,B)** and RT-PCR showed the protein **(C,D)** and mRNA **(E,F)** levels of SOX9, α-SMA, and SM22α in the VSMCs cultured with PDGF-BB in time-dependent (0, 24, and 48 h) or a concentration-dependent (0, 10, and 20 ng/mL for 24 h) manners. n = 6; **p < 0.01. **(G–I)** IF staining of SOX9 (red) and α-SMA (green) in the primary rat VSMCs cultured with or without 10 ng/mL PDGF-BB for 24 h. Nuclei were stained with DAPI (blue). SOX9 is localized predominantly in the nucleus, whereas α-SMA resides in the cytoplasm **(G)**. IF further demonstrated **(H)** and quantified **(I)** that the expression of SOX9 was highly expressed in the nuclei of VSMCs induced by PDGF-BB compared to that in the control group. n = 3; **p < 0.01; scale bar, 50 μm. **(J–N)**: VSMCs treated with 10 ng/mL PDGF-BB for 48 h enhanced their proliferative and migratory capacities compared with the control VSMCs, as evidenced by wound-healing **(J,L)**, EdU **(K,M)**, and CCK8 **(N)** assays. n = 4; **p < 0.01; scale bars, 50 μm and 100 μm.

PDGF-BB is recognized as a pivotal cytokine driving the phenotypic transformation of VSMCs. It is closely associated with several pathological processes, such as atherosclerosis and intimal hyperplasia. In this study, we detected the expression levels of VSMC markers (α-SMA and SM22α) and SOX9 in rat primary VSMCs stimulated with PDGF-BB. The expression of SOX9 increased, while the protein and mRNA levels of α-SMA and SM22α decreased in a time-dependent (0 h, 24 h, and 48 h) and concentration-dependent (0, 10, and 20 ng/mL) manner (Figures 2A–F). IF staining (Figures 2G, H) and quantification (Figure 2I) also demonstrated that in PDGF-BB-treated VSMCs, SOX9 was highly expressed and localized predominantly in the nucleus. On the contrary, SM22α, which resides in the cytoplasm, was downregulated compared to that in the control group. Moreover, treating VSMCs with 10 ng/mL PDGF-BB for 48 h significantly enhanced their proliferation and migration capacities, as evidenced by wound-healing, EdU, and CCK8 assays (Figures 2J–N). Therefore, these results indicate that SOX9 may be involved in the phenotypic transformation, proliferation, and migration of VSMCs induced by PDGF-BB.

### SOX9 knockdown inhibited the proliferation, migration, and phenotypic transformation of VSMCs

**FIGURE 3:**
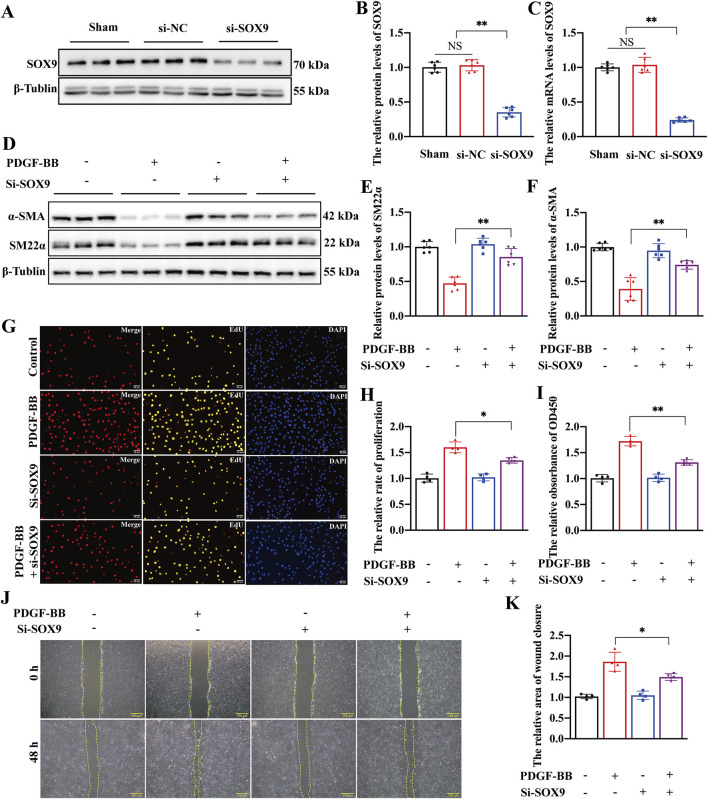
SOX9 knockdown inhibited the proliferation, migration, and phenotypic transformation of VSMCs **(A–F)** Western blot analysis **(A,B)** and RT-PCR **(C)** showed that the protein and mRNA levels of SOX9 were significantly decreased in the VSMCs transfected with si-SOX9. Compared with PDGF-BB-induced VSMCs, Western blot analysis **(D)** showed that the knockdown of SOX9 inhibited the phenotypic transformation of VSMCs, which is manifested by increased levels of SM22α **(E)** and α-SMA **(F)**. n = 6; **p < 0.01; NS indicates not significant. **(G–K)** The knockdown of SOX9 in PDGF-BB-induced VSMCs markedly inhibited their proliferation and migration capacities, as demonstrated by EdU **(G,H)**, CCK8 **(I)**, and wound-healing **(J,K)** assays. n = 4; *p < 0.05; **p < 0.01; scale bars, 50 μm and 100 μm.

We conducted a rescue experiment to investigate the role of SOX9 in the phenotypic transformation of VSMCs. First, siRNA targeting SOX9 was successfully utilized to knock down SOX9 expression at protein (Figures 3A,B) and mRNA levels (Figure 3C) in primary rat VSMCs. In addition, functional assays including wound-healing (Supplementary Figure S1A), EdU (Supplementary Figure S1B), and CCK8 demonstrated that the transfection of siRNA had no effect on the proliferation and migration of VSMCs (Supplementary Figure S1C–1E). Notably, Western blotting indicated that SOX9 knockdown significantly inhibited the phenotypic transformation of VSMCs (Figures 3D–F). Contractile marker proteins (α-SMA and SM22α) were upregulated in PDGF-BB-treated VSMCs compared to those in cells treated with both PDGF-BB and si-SOX9. In line with these findings, functional assays, including EdU, CCK8, and wound-healing assays, also showed that SOX9 knockdown markedly suppressed the proliferation and migration of VSMCs (Figures 3G–K). These results collectively highlight the critical role of SOX9 in mediating the phenotypic transformation, proliferation, and migration of VSMCs.

### SOX9 knockdown alleviates restenosis after carotid balloon injury

**FIGURE 4:**
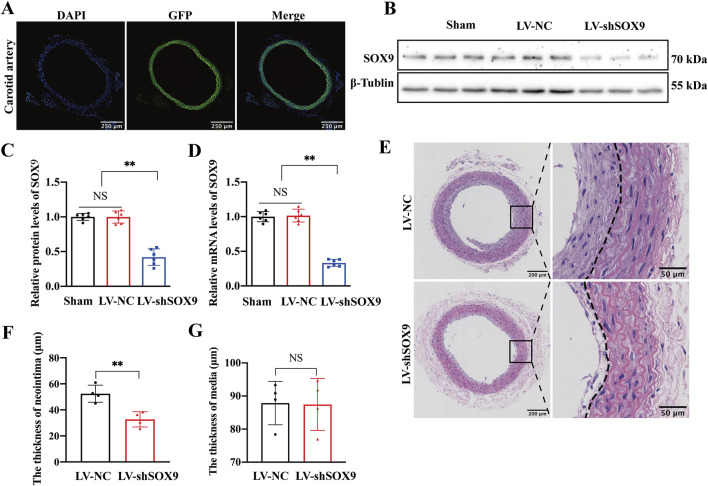
SOX9 knockdown alleviated restenosis after carotid balloon injury **(A)** GFP autofluorescence (green) was observed in LV-shSOX9-treated carotid arteries under microscopy to confirm efficient lentiviral transduction. Nuclei were stained with DAPI (blue). n = 4; scale bar, 250 μm. **(B–D)** Western blot analysis **(B)** and RT-PCR showed the protein **(C)** and mRNA **(D)** levels of SOX9 in the VSMCs transfected with LV-NC or LV-shSOX9. n = 6; **p < 0.01; NS indicates not significant. **(E–G)** Representative photomicrographs of H&E staining in the LV-NC- and LV-shSOX9-treated balloon-injured carotid arteries 14 days post-injury **(E)**. LV-shSOX9 treatment in balloon-injured carotid arteries significantly attenuated neointimal hyperplasia, as indicated by the reduced thickness of neointima after balloon injury **(F)**, while the thickness of the vascular media remained unchanged **(G)**. n = 4; **p < 0.01; NS indicates not significant; scale bars, 200 μm and 50 μm.

We employed a Pluronic gel mixed with lentivirus to knock down SOX9 expression in rat carotid arteries and investigated the role of SOX9 in carotid artery mechanical injury. As expected, GFP autofluorescence was observed in LV-shSOX9-treated carotid arteries under microscopy, confirming efficient lentiviral transduction (Figure 4A). Western blotting (Figure 4B) and RT-qPCR demonstrated that SOX9 expression was significantly decreased at both the protein (Figure 4C) and mRNA (Figure 4D) levels in LV-shSOX9-treated arteries compared to that in the LV-NC-treated group. Morphological analysis using H&E staining demonstrated that LV-shSOX9 significantly mitigated neointimal hyperplasia in balloon-injured carotid arteries, as evidenced by a marked decrease in neointimal thickness (Figures 4E,F), with no noticeable change in the medial layer (Figure 4G). These findings confirm the protective role of SOX9 downregulation in mitigating restenosis following carotid artery mechanical injury.

### SOX9 directly binds to the STAT3 promoter to mediate the phenotypic transformation of VSMCs

**FIGURE 5:**
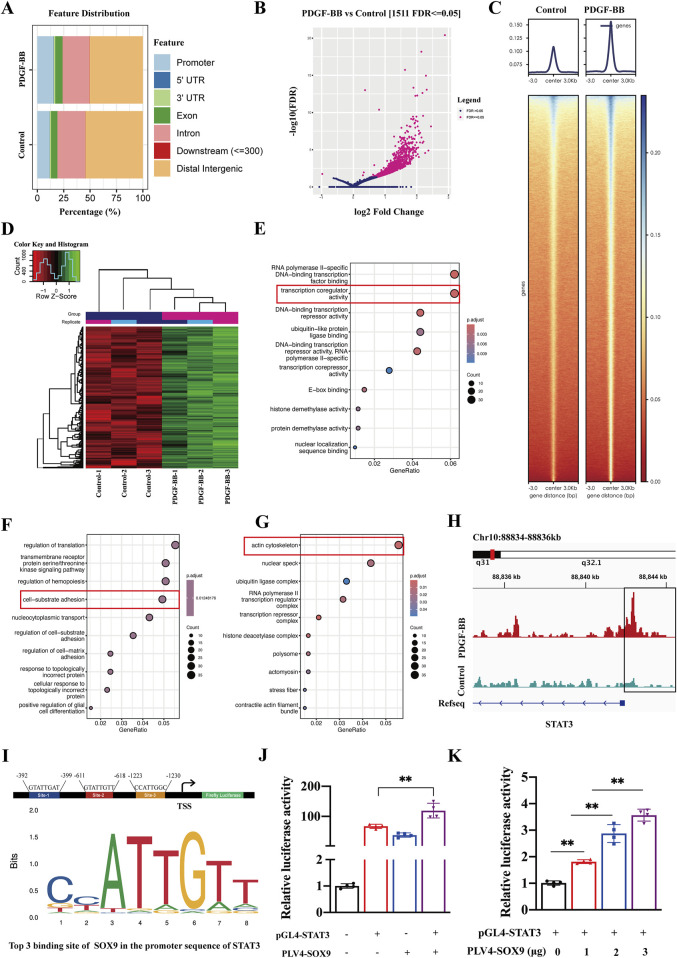
SOX9-dependent transcriptional regulation in PDGF-BB-treated VSMCs, as identified by CUT&Tag analysis. **(A)** Feature distribution of SOX9 binding sites identified by CUT&Tag in control and PDGF-BB-treated VSMCs. **(B-D)** Differential gene expression profiles of SOX9 target genes in PDGF-BB-induced VSMCs, presented as a volcano plot **(B)** and heatmaps **(C,D)**. **(E–G)** GO enrichment analysis of SOX9 target genes in molecular function **(E)**, biological process **(F)**, and cellular components **(G)** in PDGF-BB-induced VSMCs. **(H)** Integrated Genomics Viewer (IGV) tracks at lineage-specific loci for STAT3. The box represents 2 kb upstream of the TSS. **(I)** Predicted SOX9-binding motifs (site-1, site-2, and site-3) in the promoter region of STAT3, based on JASPAR analysis. **(J–K)**: The dual-luciferase reporter assay verified that the overexpression of SOX9 promoted the activity of the STAT3 promoter **(J)** in a dose-dependent manner **(K)**. n = 4; **p < 0.01.

We investigated whether SOX9, as a transcription factor, plays a role in the phenotypic dedifferentiation of VSMCs by directly binding to gene promoters following vascular injury. CUT&Tag analysis was conducted to identify the target genes of SOX9 in VSMCs. The peak distribution of SOX9 binding in both the control and PDGF-BB groups was predominantly located in the distal intergenic regions, introns, promoters, and exons (Figure 5A). The volcano plot (Figure 5B) and heatmaps (Figures 5C, D) revealed differential gene expression profiles in PDGF-BB-induced VSMCs directly targeted by SOX9. Next, we conducted Gene Ontology (GO) enrichment to explore the biological processes associated with SOX9. These genes were strongly associated with critical biological functions, including transcription co-regulator activity (Figure 5E), cell–substrate adhesion (Figure 5F), and the actin cytoskeleton (Figure 5G). All of these features are closely associated with the phenotypic transformation, migration, and proliferation of VSMCs. Interestingly, CUT&Tag analysis revealed substantial enrichment of SOX9 binding within elements located ±2 kb upstream of the transcription start site (TSS) in the STAT3 promoter (Figure 5H). The binding site prediction using the JASPAR database identified potential binding motifs for SOX9 and STAT3 (Figure 5I). Furthermore, the dual-luciferase reporter assay confirmed that SOX9 promoted the activity of the STAT3 promoter–reporter construct (Figure 5J) in a dose-dependent manner (Figure 5K). These findings highlight the direct regulatory relationship between SOX9 and STAT3, shedding light on their roles in the phenotypic transformation of VSMCs.

### AMPK mediates the SOX9–STAT3 axis in VSMCs stimulated with PDGF-BB

**FIGURE 6:**
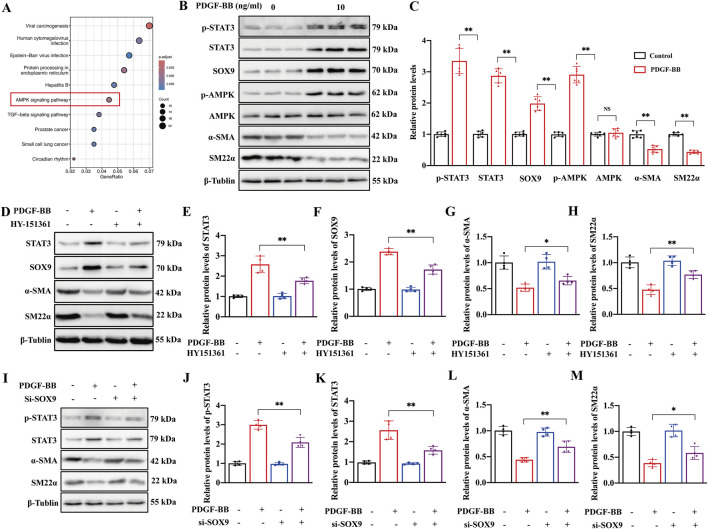
AMPK mediated the SOX9–STAT3 axis in VSMCs stimulated with PDGF-BB. **(A)** KEGG pathway enrichment map of SOX9-bound genes in PDGF-BB-treated VSMCs. **(B–C)** Western blot analysis **(B)** showed the protein levels of p-STAT3, STAT3, SOX9, p-AMPK, AMPK, α-SMA, and SM22α in the VSMCs induced by PDGF-BB **(C)**. n = 6; **p < 0.01; NS indicates not significant. **(D–H)** Western blot analysis **(D)** showed the protein levels of STAT3 **(E)**, SOX9 **(F)**, α-SMA **(G)**, and SM22α **(H)** in the VSMCs treated with DMSO, AMPK inhibitor, PDGF-BB, and PDGF-BB + AMPK inhibitor, respectively. n = 4; *p < 0.05; **p < 0.01. **(I–M)**: Western blot analysis **(I)** showed the protein levels of p-STAT3 **(J)**, STAT3 **(K)**, α-SMA **(L)**, and SM22α **(M)** in the VSMCs treated with si-NC, si-NC + PDGF-BB, si-SOX9, and si-SOX9 +PDGF-BB, respectively. n = 4; *p < 0.05; **p < 0.01.

To investigate whether the AMPK signaling pathway activates the SOX9–STAT3 axis in the PDGF-BB-induced phenotypic transformation of VSMCs, we first analyzed the Kyoto Encyclopedia of Genes and Genomes (KEGG) data; this analysis showed that PDGF-BB stimulation of VSMCs resulted in significant enrichment of SOX9 downstream genes in the AMPK signaling pathway (Figure 6A). In addition, we further found that PDGF-BB stimulation induced VSMC phenotypic transformation, as evidenced by a reduction in the expression of α-SMA and SM22α, accompanied by increased p-AMPK and elevated levels of SOX9, p-STAT3, and total STAT3. Notably, the total AMPK expression remained unchanged (Figures 6B, C). The inhibition of AMPK activation with a specific AMPK inhibitor resulted in a decrease in SOX9 and STAT3 expression, with VSMC phenotypic transformation being suppressed, as reflected by the upregulation of α-SMA and SM22α (Figures 6D–H). Moreover, we found that in si-SOX9-treated PDGF-BB-induced VSMCs, the inhibition of phenotypic transformation, as indicated by the increased expression of α-SMA and SM22α, was accompanied by a reduction in STAT3 expression (Figures 6I–M). Based on the abovementioned findings, we have identified that p-AMPK mediates the activation of the SOX9–STAT3 axis in the PDGF-BB-induced phenotypic transformation of VSMCs.

## Discussion

**FIGURE 7:**
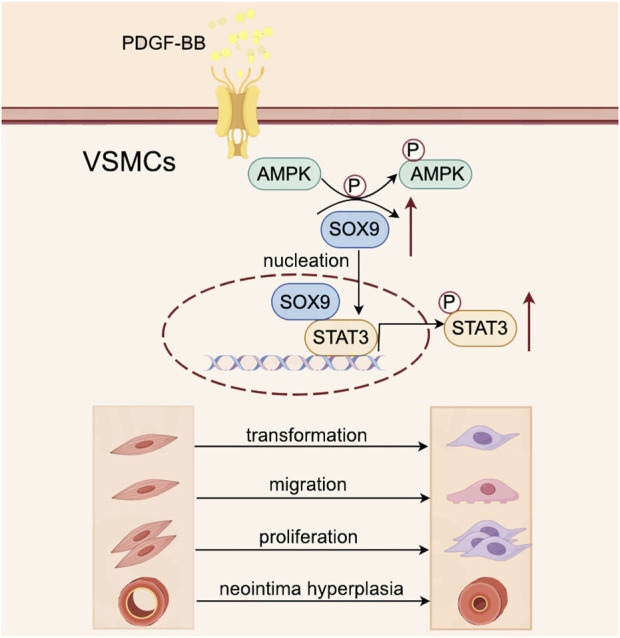
Schematic illustration of the role of the SOX9–STAT3 axis in VSMC transformation, proliferation, and migration and its contribution to neointimal hyperplasia following carotid balloon injury. PDGF-BB stimulates the phosphorylation of AMPK in VSMCs, which subsequently upregulates the expression of SOX9. Then, SOX9 translocates into the nucleus, where it directly binds to the promoter region of STAT3, leading to its activation. This signaling cascade drives VSMC transformation, proliferation, and migration, ultimately contributing to the pathological development of neointimal hyperplasia following carotid balloon injury.

The downregulation of SOX9 in VSMCs has been shown to inhibit their proliferation and phenotypic transformation, thereby mitigating neointimal hyperplasia (Deng et al., 2024), but the precise molecular mechanisms driving these effects and the regulatory interplay between SOX9 and STAT3 remain inadequately characterized. In this study, we investigated the role of the SOX9–STAT3 axis in the PDGF-BB-induced phenotypic transformation, proliferation, and migration of VSMCs. Our findings indicate that PDGF-BB stimulates AMPK phosphorylation, which subsequently upregulates SOX9 expression in VSMCs. Importantly, SOX9 was found to directly bind to the motif of the STAT3 promoter in the nucleus, thereby activating STAT3 transcription. This signaling cascade promotes the phenotypic transformation, proliferation, and migration of VSMCs, contributing to the pathological progression of neointimal hyperplasia after carotid balloon injury (Figure 7).

Following mechanical injury to the intimal layer of the carotid artery, activated platelets rapidly accumulate at the site of the lesion, triggering thrombogenesis. Upon activation, platelets recruit circulating leukocytes through their surface receptors, facilitating leukocyte recruitment to the injury site. Subsequently, activated platelets, recruited leukocytes, and inflamed endothelial cells release a series of pro-inflammatory mediators, including tumor necrosis factor-α, PDGF, fibroblast growth factor, and interleukin-6. These mediators drive the phenotypic transformation, proliferation, and migration of VSMCs and promote the synthesis of the ECM. Collectively, these processes contribute to neointimal hyperplasia and the progression of vascular restenosis (Zhao et al., 2023). Therefore, PDGF produced after vascular injury is regarded as a potent stimulator of the phenotypic transformation, proliferation, and migration of VSMCs. In this study, the protein and mRNA levels of SOX9 were upregulated in VSMCs following PDGF-BB stimulation and balloon injury, which was accompanied by the downregulation of VSMC-specific markers, such as α-SMA and SM22α. IF and IHC staining revealed that SOX9, as a transcription factor, was predominantly localized in the nucleus of VSMCs. It existed in areas with abundant phenotypic transformation of VSMCs, including the junctions between the fibrous cap and LRNC in atherosclerotic plaques. It was also abundant in the neointima following carotid artery injury. SOX9 knockdown in VSMCs exposed to PDGF-BB downregulated phenotypic transformation, proliferation, and migration of VSMCs, finally mitigating neointimal hyperplasia after mechanical injury. Furthermore, we found that PDGF-BB regulated SOX9 expression via AMPK phosphorylation. The inhibition of AMPK in VSMCs decreased SOX9 expression, which was consistent with suppressed phenotypic transformation of VSMCs. Previous studies have suggested that SOX9 modulates vascular calcification by controlling the synthesis of the ECM during the osteoblastic-like phenotypic transformation of VSMCs (Augstein et al., 2018; Faleeva et al., 2024; Chang et al., 2024) and affecting autophagy-dependent pathways that contribute to the development of transplant arteriosclerosis (Yu et al., 2022; Yu et al., 2018; Zhou et al., 2021). This study is the first to demonstrate that SOX9 also plays a significant role in regulating neointimal hyperplasia. In contrast, Augstein et al. (2018) reported that SOX9 expression in VSMCs increased after 2 h of stimulation with PDGF-BB but displayed no significant changes after 24 h of incubation. We hypothesized that this discrepancy may be due to differences in cell sources and downstream mechanisms. Additionally, variations in animal models may have contributed to the observed differences.

To date, the mechanisms driving the phenotypic transformation of VSMCs and the intricate regulatory interplay between SOX9 and STAT3 remain poorly understood. In this study, we found that compared to control VSMCs, PDGF-BB-induced phenotypic transformation of VSMCs resulted in a significant enrichment of SOX9 CUT&Tag reads at elements ±2 kb upstream of the TSS in the STAT3 promoter. This result was also confirmed by dual-luciferase reporter assays, which demonstrated that SOX9 directly binds to the STAT3 promoter, thereby enhancing STAT3 activity and the phenotypic transformation of VSMCs. Additionally, GO enrichment analysis confirmed that SOX9 is involved in transcription co-regulator activity, cell–substrate adhesion, and actin cytoskeleton organization of VSMCs, all of which are closely linked to the phenotypic transformation, migration, and proliferation of VSMCs. Previous studies (Chang et al., 2024; Han et al., 2021; Daniel et al., 2012; Calvier et al., 2017; Kurozumi et al., 2019; Kappert et al., 2006) have shown that STAT3 is involved in various types of pathological vascular changes, including vascular remodeling, vascular calcification, and pulmonary arterial hypertension. It primarily regulates the phenotypic transformation of VSMCs. Our findings contribute to understanding the role of the SOX9–STAT3 axis in mediating the phenotypic transformation of VSMCs and the development of restenosis following carotid artery injury. Elucidating the precise regulatory mechanisms of SOX9 and STAT3 in the phenotypic transformation of VSMCs is critical for developing effective preventive and therapeutic strategies to overcome ISR.

The current study has several limitations. We focused on contractile phenotype markers (SM22α and α-SMA) to assess VSMC phenotypic transformation; however, evaluating other synthetic markers could provide additional insights. Additionally, we did not explore the impact of the SOX9–STAT3 axis on extracellular matrix components, which are crucial in VSMC phenotypic modulation and vascular remodeling. Our study utilized a carotid balloon injury model with lentiviral-mediated SOX9 knockdown; however, future studies incorporating lineage tracing and SOX9 knockout mice would offer a more comprehensive understanding of phenotypically transformed VSMCs in the neointima following mechanical injury.

In conclusion, this study demonstrated that PDGF-BB induces the phenotypic transformation, proliferation, and migration of VSMCs through the p-AMPK pathway, which promotes the nuclear translocation of SOX9. SOX9 directly binds to the STAT3 promoter motif in the nucleus, thereby upregulating STAT3 expression and driving the phenotypic transformation of VSMCs. The inhibition of the SOX9–STAT3 axis effectively suppressed the transformation, proliferation, and migration of VSMCs, leading to decreased neointimal hyperplasia following mechanical injury. Targeting SOX9 presents a promising therapeutic strategy for the treatment of ISR.

## Data Availability

The original contributions presented in the study are included in the article/Supplementary Material; further inquiries can be directed to the corresponding authors.

## Supplementary material

SUPPLEMENTARY FIGURE S1 siRNA transfection had no effect on the proliferation or migration of VSMCs. **(A–E)**: The siRNA targeting SOX9 was successfully used to knock down SOX9 expression, and functional assays, including wound-healing **(A)**, EdU **(B)**, and CCK8, demonstrated that siRNA transfection had no effect on the proliferation and migration of VSMCs **(C–E)**. n=4; NS indicates not significant; scale bar: 50 μm and 100 μm.
