# Supplementary figures and images for: SOX9 mediates the phenotypic transformation of vascular smooth muscle cells in restenosis after carotid artery injury
Source: Front Cell Dev Biol. 2025 Jun 18;13:1592594. doi: 10.3389/fcell.2025.1592594 (PMC12213809; doi:10.3389/fcell.2025.1592594)

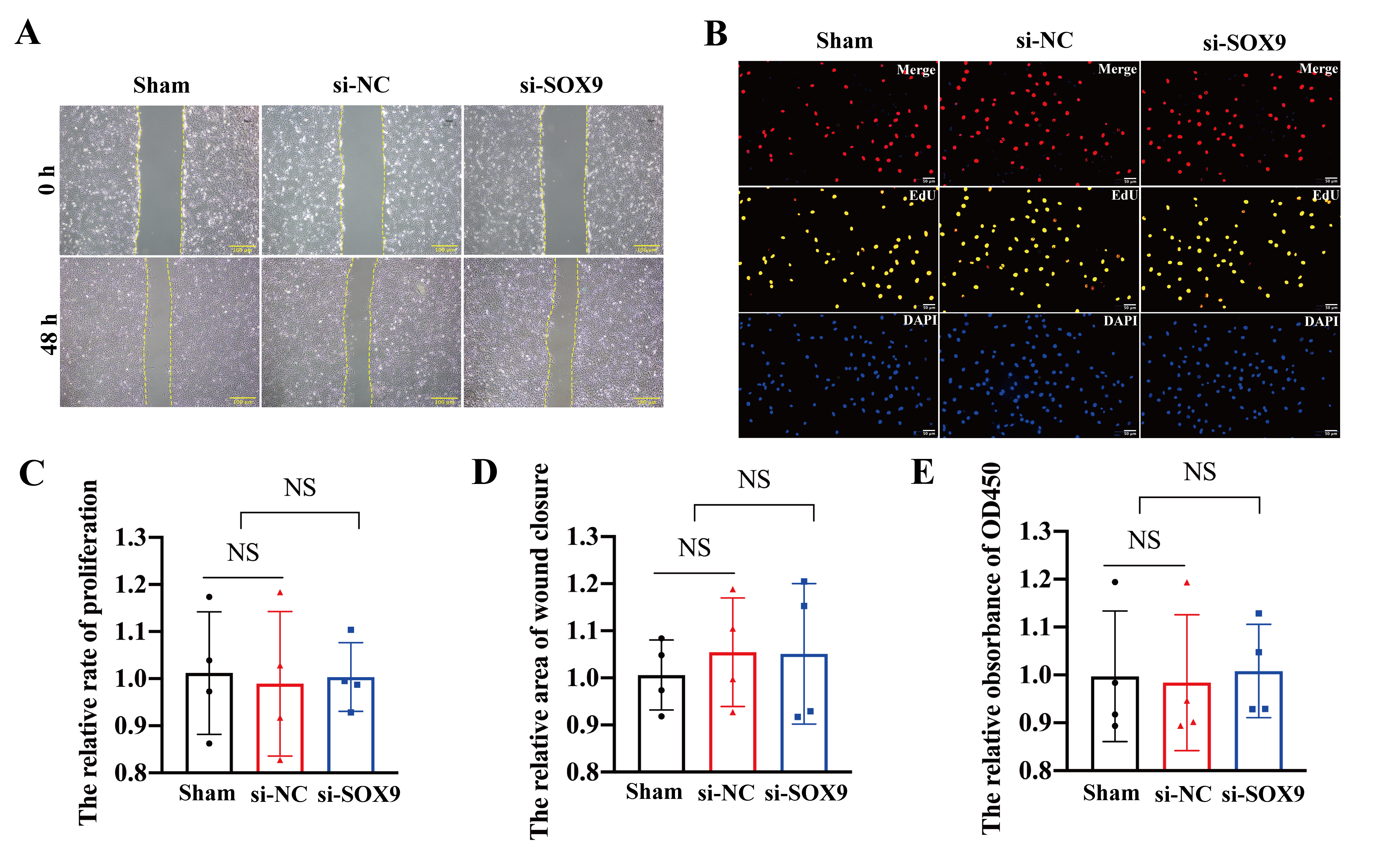

Supplement: Supplementary file 1 [file Image1.jpeg]
